# Sarcome de kaposi penien chez un patient seronegatif

**DOI:** 10.11604/pamj.2017.28.61.13248

**Published:** 2017-09-21

**Authors:** Mohammed Alae Touzani, Othmane Yddoussalah

**Affiliations:** 1 Université Mohammed 5, Faculté de Médecine et de Pharmacie de Rabat, Hopital Ibn Sina, Service d’Urologie B, Maroc

**Keywords:** Cancer pénien, Kaposi, VIH, Tumor of the penis, kaposi, HIV

## Abstract

Sarcomas of the penis account for less than 5% of all tumors of the penis. They are dominated by Kaposi's sarcoma that mainly affects HIV-positive patients. However, recent studies have shown a relationship between Kaposi's sarcoma and HHV-8 infection (Human herpes virus-8), which explains why this sarcoma occurs in non-immunocompromised and HIV-seronegative patients. We here report the case of a 72-year old patient, with no previous medical history, reporting of gradual onset of tumor-like granulation tissue of 3 years duration at the level of the gland, without secondary location. Given the patient's clinical condition, epidermoid carcinoma or sarcomatoid carcinoma of the penis were suspected. Initial biopsy was negative, the second was in favor of Kaposi's sarcoma, confirmed by immunohistochemistry. The patient underwent chemotherapy.

## Image en médecine

Les sarcomes péniens représentent moins de 5% des tumeurs péniennes. Elles sont dominées par le sarcome de Kaposi que l'on retrouve le plus souvent chez les sujets HIV positifs. Cependant, de récentes études ont montrées une association entre le sarcome de Kaposi et l'infection par le HHV-8 (Human Herpes Virus 8), ce qui justifie l'occurrence de ce sarcome chez les patients non immunodéprimés, non séropositifs. Nous rapportons ici le cas d'un patient de 72 ans, sans antécédents, qui rapporte depuis 3 ans l'apparition progressive d'un bourgeon charnu d'allure tumorale au niveau du gland, sans autre localisation. Devant le tableau clinique, nous avons suspecté un carcinome épidermoïde ou un carcinome sarcomatoide de la verge. Une première biopsie faite est revenue négative, la seconde est revenue en faveur d'un sarcome de Kaposi, confirmé par l'immunohistochimie. Le patient a bénéficié d'une chimiothérapie.

**Figure 1:**
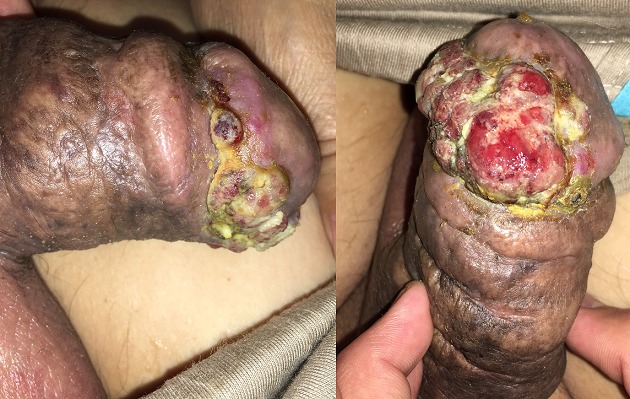
Sarcome de kaposi

